# Personalised Risk Modelling for Older Adult Cancer Survivors: Combining Wearable Data and Self-Reported Measures to Address Time-Varying Risks

**DOI:** 10.3390/s25072097

**Published:** 2025-03-27

**Authors:** Zoe Valero-Ramon, Gema Ibanez-Sanchez, Antonio Martinez-Millana, Carlos Fernandez-Llatas

**Affiliations:** 1 ITACA-SABIEN, Universitat Politècnica de València, 46022 Valencia, Spain; 2 Department of Clinical Science, Intervention and Technology, Karolinska Institutet, 171 77 Stockholm, Sweden

**Keywords:** process mining, wearable devices, time-varying risks, older adult cancer patients, dynamic risk models

## Abstract

Recent advancements in wearable devices have significantly enhanced remote patient monitoring, enabling healthcare professionals to evaluate conditions within home settings. While electronic health records (EHRs) offer extensive clinical data, they often lack crucial contextual information about patients’ daily lives and symptoms. By integrating continuous self-reported outcomes related to vulnerability, anxiety, and depression from older adult cancer survivors with objective data from wearables, we can develop personalised risk models that address time-varying risk factors in cancer care. Our study combines real-world data from wearable devices with self-reported information, employing process mining techniques to analyse dynamic risk models for vulnerability and anxiety. Unlike traditional static assessments, this approach recognises that risk factors evolve. Collaborating with healthcare professionals, we analysed data from the LifeChamps study to create two dynamic risk models. This collaborative effort revealed how activity and sleep patterns influence self-reported vulnerability and anxiety among participants. It underscored the potential of wearable sensors and artificial intelligence techniques for deeper analysis and understanding, making us all part of a larger effort in cancer care. Overall, patients with prolonged sedentary activity had a higher risk of vulnerability, while those with highly dynamic sleep patterns were more likely to report anxiety and depression. Prostate-metastatic patients showed an increased risk of vulnerability compared to other cancer types.

## 1. Introduction

In recent years, advancements in wearable technology have transformed the healthcare landscape, enhancing health monitoring and revolutionising patient–provider interactions, especially in remote care. Wearable devices, such as smartwatches and fitness trackers, continuously collect physiological data (heart rate, activity levels, and sleep partners, among others) that provide real-time insights into the health status. Integrating these technologies into patient care represents a shift from traditional episodic assessments to real-time monitoring, providing a comprehensive understanding of an individual’s health status [1]. This approach represents a shift from traditional static assessments to real-time, dynamic monitoring, providing a comprehensive understanding of how risk factors evolve over time [1].

Wearable technology also facilitates the early detection of health issues by identifying subtle changes in baseline metrics, such as a gradual increase in heart rate [2]. Additionally, these devices empower patients to take a proactive approach to their health by providing immediate feedback and encouraging healthier lifestyles [3]. The data generated can inform treatment plans and optimise healthcare strategies, allowing for tailored interventions and improved patient outcomes [1,4].

Research on wearable devices has highlighted their benefits and limitations. For example, a systematic review and meta-analysis [5] explored the use of activity trackers in older adults, emphasising the need for further research in clinical practice and home-based monitoring. Moreover, older adults with cancer often face a unique set of challenges that can contribute to feelings of vulnerability and anxiety, frailty, and other age-related changes [6]. Measuring and analysing self-reported and objective data are essential steps for cultivating a comprehensive understanding of these challenges. Self-reported information offers valuable insights into patients’ subjective experiences, including their perceived vulnerability, anxiety, and the emotional impact of their cancer journey.

Incorporating real-world data from wearable devices into clinical data presents a promising opportunity to enhance cancer patient care. While cancer patients undergo extensive evaluations and treatment regimens documented in their electronic health records (EHRs), these records often lack contextual information about their daily life, activity levels, and symptoms that significantly impact their cancer journey [7].

Integrating data from wearables, mobile applications, and patient-reported outcomes can provide a more comprehensive view of the patient’s experience. Older adults with cancer face unique challenges, including vulnerability and anxiety, making it essential to analyse both self-reported and objective data. Self-reported insights into perceived vulnerability and emotional impact are particularly valuable.

Several studies have explored wearable devices for managing cancer survivors’ health. For instance, Rossi et al. (2018) [8] examined the acceptability and validity of a physical activity tracker for endometrial cancer survivors, a group with high obesity rates. Another study focused on colorectal and endometrial patients, using wearables to change physical activity behaviour [9]. A recent review highlighted the integration of wearables in prostate cancer research, assessing daily activity and adherence to exercise interventions [10]. While these studies underscore the importance of wearables for monitoring activity and exercise adherence, they often lack a temporal analysis of these factors.

The rise of wearable health monitoring devices generates a wealth of continuous, real-world patient data, but extracting meaningful insights requires effective analysis. Traditional methods often struggle with the complexity of these datasets, while advanced analytics techniques, particularly those using artificial intelligence (AI), can uncover patterns that manual reviews might miss [11]. Traditional prediction methods typically focus on demographic and clinical characteristics [4], often overlooking the temporal aspect of disease progression [12]. There is growing interest in applying AI in healthcare, but many AI techniques lack explainability, affecting healthcare professionals’ trust. Explainable AI (XAI) and interactive models seek to build trust between doctors and AI systems [13].

Interactive process mining [14] offers methods to extract knowledge from data stored in event logs generated by health information systems and real-world data sources like sensors. This approach can model individual behaviours and stratify patients based on clinical and real-world data [15]. By combining continuous data with advanced analytics, process mining facilitates the dynamic assessment of disease processes and individual risk profiles.

Few studies have integrated real-world data from wearable devices with self-reported data to develop personalised risk models for cancer. For example, one study [16] introduced a cancer-specific predictive model identifying features linked to poor prognosis in patients with non-small cell lung cancer, breast cancer, and non-Hodgkin lymphoma using AI, clinical data, questionnaire responses, and wearable data. Another review discussed the benefits of activity monitoring devices in categorising physical activity levels and health outcomes for lung cancer patients [17]. Additionally, another study [18] applied machine learning algorithms to analyse self-reported physical activity and heart rate data, assessing cardiovascular disease-related metrics.

Despite these contributions, further research is necessary to explore the incorporation of time-varying perspectives into risk models, leveraging data from wearable devices and the analytical potential of AI techniques. Integrating self-reported outcomes and objective data from wearable devices could significantly enhance the development of personalised risk models that account for underlying time-varying risk factors.

This study aims to integrate wearable technology and self-reported measures to develop personalised risk models for older adult cancer survivors, focusing on time-varying risk factors. Using process mining techniques and collaborating with healthcare professionals, this study analyses data and develops dynamic risk models for vulnerability and anxiety among older adult cancer survivors. This study is related to challenges C8, C4 and C1 of the Process Mining in Healthcare Manifesto [19]. Unlike static assessment methods, this approach allows us to account for time-varying risk factors and measure their impact on self-reported vulnerability and anxiety. We analysed data collected during the LifeChamps study and collaborated with healthcare professionals (HCPs) to co-design and develop two dynamic risk models. Our results reveal how activity and sleep patterns influence self-reported vulnerability and anxiety among the study population.

The subsequent sections of this paper explore the various layers of our investigation. We start with the background of our study, relating to older adult cancer survivors. After this, we describe the materials, methods, and dataset used to build the results. Then, this work presents the results achieved in the inquiry. Following this, we delve into discussing the results and their limitations. Finally, we present our conclusions.

## 2. Background: Older Adult Cancer Survivors

Older cancer survivors face unique challenges stemming from their age and specific characteristics, including multimorbidity, frailty, and the psychosocial impacts of cancer. These factors can complicate treatment and overall care, while age-related changes in physiology and cognitive function may hinder their ability to cope with cancer treatment and recovery. The 2021 report from the International Society of Geriatric Oncology (SIOG) identified key priorities for advancing care for older cancer patients globally [20]. This collaborative effort highlighted the need for shared decision-making between healthcare providers and older patients and a multidimensional evaluation of their physical, cognitive, psychological, social, and functional status. Collecting and analysing real-world data from passive monitoring technologies can give HCPs a holistic view of older adult cancer patients’ functional status. Additionally, continuous data can support advanced analytics and predictive modelling, empowering healthcare teams to make informed, data-driven decisions [21].

Older adults with cancer face unique challenges that contribute to feelings of vulnerability and anxiety. They often have decreased physiological reserves and an increased risk of treatment-related toxicity due to multimorbidity, frailty, and age-related changes [6]. This heightened vulnerability can lead to concerns about functional decline and recovery from cancer treatments. Additionally, the cancer diagnosis itself can be profoundly stressful, with older patients worrying about its impact on their quality of life and independence and the potential burden on their loved ones.

These feelings of vulnerability and anxiety significantly affect the well-being and treatment outcomes of older cancer survivors [22]. Measuring these issues through self-reported data is crucial, as it provides insights into patients’ perceived vulnerability and emotional impact. Validated assessment tools, like patient-reported outcome measures (PROMs), can effectively capture these subjective experiences [23].

A notable relationship exists between anxiety disorders and sleep quality; older adults with anxiety often experience poor sleep, making sleep efficiency—defined as the percentage of time spent sleeping compared to the time in bed—an essential focus. A sleep efficiency below 80% is a significant mortality risk, while a sleep efficiency of 90% or above offers substantial health benefits [24]. Additionally, anxiety and physical activity levels are interconnected, where anxiety can lead to sedentary behaviour and low physical activity can exacerbate anxiety symptoms [25]. Understanding the relationship between vulnerability and physical activity is vital for improving health outcomes in older adults. Regular physical activity enhances physical function and quality of life during and after cancer treatment [26].

Further research is essential to integrate a time-varying perspective into risk models by utilising data from wearable devices. This approach requires researchers to consider how health metrics change over time rather than relying on static snapshots of a patient’s health. Wearable devices provide continuous data on various health parameters, such as activity levels and sleep patterns, allowing for a more nuanced understanding of health evolution, which is crucial for accurate risk assessment. For example, fluctuations in physical activity or sleep quality can significantly affect a cancer survivor’s vulnerability to anxiety and depression. Researchers can develop personalised risk models by combining self-reported outcomes—like feelings of vulnerability and anxiety—with objective data from wearables. These models would identify underlying time-varying risk factors impacting older adult cancer survivors.

## 3. Materials and Methods

This work aimed to integrate real-world data from wearable devices with self-reported information and analyse them using process mining techniques to evaluate dynamic risk models for older adult cancer survivors. This section outlines the materials and methods used to achieve these objectives, detailing the interactive process mining paradigm for developing the dynamic risk models and describing the dataset and study conducted. The study was part of the European Union’s Horizon 2020 project, LifeChamps, which seeks to create a collective platform to support cancer champions.

### 3.1. Interactive Process Mining in Place

Analysing data from wearable devices and reported outcomes is essential to enhancing understanding of healthcare processes. This comprehensive analysis can reveal trends, identify potential improvements, and inform decision-making. Therefore, the involvement of healthcare experts in this process is crucial, as their expertise ensures that the data are interpreted accurately and applied effectively to optimise patient care and outcomes.

Interactive process mining (IPM) [14] is a flexible methodology that enables professionals to gain insights into health processes over time. Unlike traditional methods that visualise isolated data from a single individual—often failing to provide a deeper understanding—this approach allows for a comprehensive analysis of patient behaviour in context. Its adaptability supports the application of precision medicine in a more personalised manner, empowering experts to refine models in tandem with the evolving behaviours of patients.

Within the IPM methodology, an Indicator refers to any information that aids in understanding or measuring the characteristics or intensity of a particular phenomenon, as well as evaluating its evolution over time. Consequently, a Process Indicator (PI) is a specific representation of a process that serves as an indicator for assessing or measuring the behaviour of that process [14].

Also, in the context of IPM, a formal procedure was established to develop Interactive Process Indicators (IPIs), a Data-Rodeo. Based on a high-level description, a Data-Rodeo describes how process mining (PM) and healthcare experts interact until an IPI is wholly built.

### 3.2. Dynamic Risk Models

Our study aimed to explore the development of time-varying risk models by combining self-reported and objectively monitored data. With this goal in mind, our work is supported by the concept of dynamic risk models. The work presented in [27] proposes an interactive and question-based methodology for deploying dynamic risk models for chronic conditions using process mining. This formal methodology incorporates clinical experts’ needs following the IPM methodology [14] to provide understandable results, considering the nature of the chronic disease during the formulation. Following this methodology, experts play a crucial role in co-developing dynamic risk models and tracking the evolution of related variables.

Although the methodology was proposed for chronic conditions underlying processes, it can also be used for other diseases, such as cancer. Some studies have demonstrated the feasibility of its application in health domains. Examples include supporting chronic disease management [15], understanding prostate cancer using prostate-specific antigen (PSA) values [28], and a dynamic behavioural approach to nutritional assessment using process mining [29].

The methodology provides a clear framework for applying IPM to analyse the underlying processes using time-varying variables. Clinical experts can leverage this methodology to engage deeply with patients’ conditions by formulating pertinent questions and validating or refining the results. Meanwhile, process mining experts enhance their understanding of relevant techniques. The methodology comprises six key steps: (1) defining the specific question; (2) analysing risk factors and variables relevant to the question; (3) verifying the quality and availability of data; (4) formalising the IPI associated with the question; (5) conducting stratification analysis; and (6) validating the results with field experts.

Process mining enables the construction of individual and human behaviour models [30], which leads to the creation of the IPI associated with the question in the fourth step of the methodology. This approach facilitates the analysis of health determinants the variability and progression of a disease over time. The fifth step of the methodology deals with the stratification analysis, employing clustering techniques. These unsupervised data mining methods group traces exhibiting similar behaviours while maximising distinctions between different groups. They can be viewed as a process mining conformance technique, as they utilise distances between models to organise the traces [31]. This dynamic approach to stratification enhances our understanding of clinical cases by revealing meaningful patterns and relationships within the data. Concretely, with an aligned heuristic distance [32], we used the Quality Threshold Clustering (QTC) [33] algorithm. This algorithm requires a quality threshold to determine the maximum distance among traces in the cluster.

### 3.3. LifeChamps Intervention

This work was conducted as part of the LifeChamps project, an EU Horizon 2020 initiative to develop a digital platform to monitor health-related quality of life and frailty in cancer patients aged 65 and older. The LifeChamps digital care platform (LC platform) includes several components, including a mobile app for collecting patient-reported outcome measures (PROMs), providing educational content, delivering meditation and mindfulness exercises, and offering personalised motivational messages through a Health Recommender System. Additional features include personal sensors, an in-home location system, AI-based clinical monitoring algorithms, a digital clinical dashboard for HCPs, and a Big Data infrastructure for managing diverse data from both patients and clinical settings. The project aims to gather multi-modal data from PROMs, clinical records, and sensor inputs to enhance patient outcomes, particularly in follow-up geriatric oncology care after cancer treatment and recovery. Following the protocol outlined in [34], the LifeChamps study is a feasibility study conducted across four sites: the Academic Primary Health Care Centre (Sweden), Aristotle University of Thessaloniki (Greece), Medical Research Institute of Hospital La Fe (Spain), and the University of Glasgow (Scotland), each enrolling older adult cancer survivors independently to evaluate the LC platform.

Patients participating in the pilots were provided with a smartwatch (Activity tracker wristband Fitbit Charge 4), an intelligent weight scale (Withings Body+), and motion sensors installed in patients’ homes (https://www.mysphera.com/locs/, accessed on 11 December 2024) to monitor basic physiological parameters and activity. The Fitbit Charge 4 incorporates a 3-axis accelerometer, an optical heart rate monitor, GPS, a vibration motor, NFC, and an altimeter used to record some health features, such as sleep or fitness. The Withings Body+ is a precision smart scale that provides detailed information about body fat, muscle mass, and total body water. The motion sensors installed in patients’ homes, LOCS, included sensors that recorded movement, humidity and temperature and sensors on the doors that recorded entrances and exits.

The LC platform includes a digital clinical dashboard for HCPs, referred to as the LC Dashboard. This analytical tool is designed to assist HCPs in monitoring cancer patients’ progress using process mining techniques. The dashboard aggregates data from various sources to create and analyse IPIs, providing valuable insights into the processes affecting cancer patients involved in the LifeChamps project. The LC Dashboard supports the development of IPIs and dynamic risk models, offering dynamic views that reflect the current status of patient processes. Its analytical capabilities allow for multiple perspectives on the data, enhancing the understanding of the specific patient population. Our methodology employed the LifeChamps version of PMApp [35] as the chosen process mining tool.

### 3.4. LifeChamps and Its Variables

Based on the objectives and considerations of the LifeChamps intervention, our study concentrated on two primary perspectives for analysis using dynamic risk models: self-reported vulnerability and anxiety/depression. The aim was to explore the relationships between these two concepts and the data collected from wearable devices from a temporal perspective.

As previously noted, anxiety disorders can significantly impact sleep quality. Therefore, we included sleep efficiency—measured by the activity tracker—as a critical time-varying variable in our study. Sleep efficiency values ranging from 0.8 (80%) to 0.89 (89%) indicate normal sleep. While this range is generally acceptable for older adults, it is not considered optimal. In contrast, a sleep efficiency of 0.9 (90%) or higher is categorised as beneficial sleep; this level is believed to confer the most significant health advantages for older adults [36]. Additionally, given the established link between physical activity levels and anxiety and vulnerability, we considered the activity levels reported by the activity tracker as another critical time-varying variable in our analysis. Looking into the literature [37], we considered a sedentary day as one during which fewer than 5000 steps were registered, medium activity as between 5000 and 10,000 steps being registered, and an active day as one during which more than 10,000 steps were registered.

Consequently, based on this evidence and the available information within the LifeChamps intervention, the time-varying risk factors to be studied were sleep and physical activity, which were combined with the self-reported data to develop the corresponding dynamic risk models. To investigate the relative danger of sleep and physical activity in anxiety and vulnerability, we used the relative risk (RR), also sometimes known as the risk ratio, the probability of an event occurring in the exposed group versus the likelihood of the event occurring in the non-exposed group [38]. This is calculated as follows: (Probability of an event in an exposed group)/(Probability of an event in a not exposed group).

**Theorem** **1** (Relative Risk). *The relative risk (RR) is a measure used to compare the probability of an event E occurring in an exposed group A to the probability of the same event occurring in an unexposed group ¬A. It is defined as*

$$RR=\frac{P(E|A)}{P(E|\neg A)},$$

*where*

- *P(E∣A): The probability of the event E occurring in the exposed group A,*
- *P(E∣¬A): The probability of the event E occurring in the unexposed group ¬A.*

*Given the following definitions,*

$$\begin{array}{cc} a\; & :\;\mathrm{Number}\;\mathrm{of}\;\mathrm{individuals}\;\mathrm{in}\;\mathrm{the}\;\mathrm{exposed}\;\mathrm{group}\;\mathrm{who}\;\mathrm{experience}\;\mathrm{the}\;\mathrm{event},\\ b\; & :\;\mathrm{Number}\;\mathrm{of}\;\mathrm{individuals}\;\mathrm{in}\;\mathrm{the}\;\mathrm{exposed}\;\mathrm{group}\;\mathrm{who}\;\mathrm{do}\;\mathrm{not}\;\mathrm{experience}\;\mathrm{the}\;\mathrm{event},\\ c\; & :\;\mathrm{Number}\;\mathrm{of}\;\mathrm{individuals}\;\mathrm{in}\;\mathrm{the}\;\mathrm{unexposed}\;\mathrm{group}\;\mathrm{who}\;\mathrm{experience}\;\mathrm{the}\;\mathrm{event},\\ d\; & :\;\mathrm{Number}\;\mathrm{of}\;\mathrm{individuals}\;\mathrm{in}\;\mathrm{the}\;\mathrm{unexposed}\;\mathrm{group}\;\mathrm{who}\;\mathrm{do}\;\mathrm{not}\;\mathrm{experience}\;\mathrm{the}\;\mathrm{event}, \end{array}$$

*the probabilities can be expressed as*

$$P\left(E|A\right)=\frac{a}{a+b},\;P\left(E|\neg A\right)=\frac{c}{c+d}.$$

*Thus, the relative risk can be calculated as*

$$RR=\frac{\frac{a}{a+b}}{\frac{c}{c+d}}=\frac{a(c+d)}{c(a+b)}.$$

An RR greater than 1.0 indicates a positive association or an increased risk of developing a health outcome. An RR of 1.0 suggests that exposure does not affect the outcome, while an RR less than 1.0 indicates that exposure decreases the risk, acting as a protective factor. The 95% confidence interval provides an estimate of the RR. Suppose the RR exceeds 1.0, and the confidence interval does not include 1. In that case, it suggests that events are significantly more likely to occur in the exposed group compared to the other group [39].

### 3.5. Data Source and Study Population

In this work, we used the LifeChamps real-life feasibility pilot dataset [34], which included 121 unique cancer survivors’ data from the wearable and sensors specified, among others (see Table 1 for more details). The real-life feasibility pilot occurred between January 2023 and November 2023, during which patients were monitored for three months. Following the requirements of the European Union and state legislation, the Declaration of Helsinki, the Oviedo Convention on Human Rights and Biomedicine, and the European Code of Conduct for Research Integrity, this protocol was submitted for ethical approval for all four study sites: Swedish Ethical Review Authority with Registration No. 2022-00562-01, Greek Research Ethics & Deontology Committee with Registration No. 267203/2022, Ceim Hospital Universitario y Politécnico la Fe in Spain with Registration No. 2019-157-1, and East of Scotland Research Ethics Committee HRA with Registration No. INGN21HS566. Following ethical committee procedures, the documentation presented included a Data Protection Impact Assessment (DPIA) to identify and mitigate any data protection-related risks arising from the study, which may affect both the organisations and individuals it engages with. Furthermore, a Joint Controller/Data Sharing Agreement was signed by all involved parties.

All participants were given a study summary leaflet, participant information sheet (PIS), and consent form to read and sign if interested in taking part. The PIS made it explicit that LifeChamps is not a medical device and does not replace standard care, and in the case of any issues or emergencies, patient participants should always contact their clinical team. The PIS also confirmed that patients deciding to decline at the consent stage or withdraw after providing consent would have no impact on the treatment or care services they received from their clinical team.

Table 2 and Table 3 show a general descriptive overview of the characteristics of the participants per country. Overall, the mean age was 71.63 ± 5.93 years old, and a total of 58 (almost 48%) of the participants were women, which indicates a good balance between women and men in the study.

This study included cancer survivors and HCPs, who were invited to participate in the co-creation of results following the methodology outlined in Section 3.2. Specifically, 36 HCPs contributed to developing and validating the findings. Table 4 provides a demographic overview of the HCPs across the pilot sites.

As explained, the LifeChamps intervention included a set of sensors to be used by patients or installed in their homes, specifically an activity tracker, a smart scale, and in-home motion sensors. Insights about the data being collected in the context of the real-life pilot feasibility trials and after being analysed in the context of the LC Dashboard are included in Table 1.

One of the LifeChamps study goals was to look for insights into changes in participants’ quality of life, collecting and analysing some self-perceived parameters. With this in mind, two dimensions were proposed for analysis, namely anxiety and depression on the one hand and vulnerability on the other, as the two indicators for quality of life and frailty in the studied population.

The Patient Health Questionnaire-4 (PHQ-4) was developed and validated to address the fact that anxiety and depression are two of the most prevalent and frequently co-morbid illnesses among the general population. The PHQ-4 has four items answered on a four-point Likert-type scale. Its purpose is to allow for the ultra-brief and accurate measurement of core symptoms/signs of depression and anxiety by combining the two-item measure (PHQ-2), consisting of core criteria for depression, and a two-item measure for anxiety (GAD-2) [40]. In this work [40], PHQ-4 scores were operationally categorised as normal (0–2), mild (3–5), moderate (6–8), and severe (9–12). The Vulnerable Elders Survey (VES-13) is a simple function-based tool for screening older adults at risk of health deterioration. The components of the 13-item questionnaire include age, self-rated health, limitations in physical function, and disability. Its score ranges from 0 to 10. The lower the score, the better [41]. In this work [41], VES-13 scores were categorised as not vulnerable for scores 0–2 and vulnerable for scores of 3+ by the authors.

## 4. Results

This section presents the results designed and developed in the context of the LifeChamps study following a co-creation process with clinical experts in the cancer field and using data from real-life pilots.

We conducted 12 working sessions with the 36 HCPs participating across four pilot sites, focusing on changes in participants’ quality of life through self-perceived parameters. HCPs chose anxiety/depression and vulnerability as the dimensions for analysis. Below is a summary of the three co-creation sessions:

- Session 1. Clinical experts discussed their interests in analysing anxiety/depression and vulnerability within the LifeChamps context. It was decided to combine self-reported patient data with objective data from activity trackers and smart scales. The session also introduced analysis tools, allowing experts to familiarise themselves with preliminary results. Process mining experts gained clinical insights to develop initial findings using the PHQ-4 and VES-13 questionnaires.
- Session 2. Preliminary results were presented, and data curation was conducted with clinical experts. Feedback indicated a desire to include RR computations in the analysis. Experts requested the addition of various dynamic risk variables, including weight, body composition, activity levels, sleep metrics, cancer type, and age. The team also discussed appropriate cut-off points for these measures.
- Sessions 3. The final sessions showcased refined results and trained clinical experts on the tool’s functionalities. The analysis incorporated the Body Mass Index (BMI), sleep efficiency, and daily activity (steps). The main goal was to empower experts to analyse results and derive relevant clinical insights autonomously.

We adhered to the methodology outlined in Section 3 to develop the dynamic risk models by applying process mining techniques to the log created from the data sources detailed in Table 1. Concretely, patients were asked to fill out questionnaires once per month during the three months of the study, plus one at the beginning.

Process mining uses recorded action logs to extract and visualise workflows, making processes more understandable. In this diagram, data are structured as event logs, where each event corresponds to a case (a single patient), an activity (e.g., daily steps), and a specific point in time. A set of events for one patient forms a case, while multiple cases create a log.

**Figure 1:**
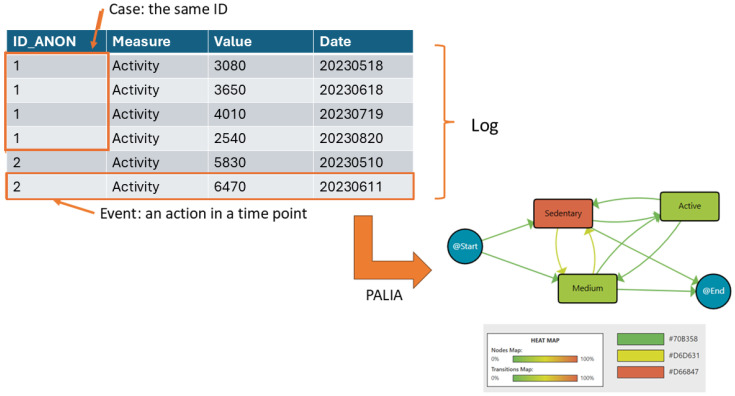
Process Mining rationale.

On the left side of Figure 1, the log is represented as a table. Each row is an event, showing the patient ID (ID_ANON), the recorded activity (daily steps), the value (number of steps), and the date when the activity was recorded. Events from the same patient are grouped under the same ID. Then, this log is analysed using the PALIA algorithm after the recorded daily steps are discretised into three categories: sedentary (fewer than 5000 steps), medium (between 5000 and 10,000 steps), and active (more than 10,000 steps). The workflow on the right side visualises the extracted patterns. Nodes represent different activity levels (sedentary, medium, active, and start/end). The heat map colouring indicates the median time spent in each activity. Green nodes reflect shorter durations, while red nodes indicate longer durations. Arrows mean the transition between one activity and the following one. The colour intensity of the arrows reflects the frequency of these transitions. For example, a more intense red arrow indicates a higher frequency of patients moving between those states. Then, this workflow summarises the sequence of activity levels, the duration spent in each state, and the frequency of transitions between them. For example, it can be observed that patients frequently transition between medium and sedentary. Also, a significant amount of time being spent in the sedentary state is indicated by its reddish node colour, while transitions to and from the active state being less frequent is indicated by lighter-coloured arrows.

The heat map legend at the bottom assists in the interpretation of the colour intensities of nodes (median duration) and transitions (frequency). This workflow provides insights into patients’ activity patterns, helping to identify trends and areas for intervention.

### 4.1. Vulnerability Dynamic Risk Model

This dynamic risk model combines self-reported vulnerability measures using the VES-13 with objective information from the activity tracker. We considered that there was “No vulnerability Risk (0)” when none of the VES-13 questionnaires were positive (scores < 3) and “Vulnerability Risk (1)” when at least one VES-13 questionnaire was positive (score ≥ 3). It is worth mentioning that three VES-13 questionnaires were collected for all participants during the LifeChamps study.

Working with clinicians in the different sessions, we analysed the activity level reported by the activity tracker with the number of steps per day to discover groups of activity behaviour as the time-varying risk variable. As explained in Section 3, we considered a sedentary day as one where fewer than 5000 steps were registered, medium activity as between 5000 and 10,000 steps being registered, and an active day as one where more than 10,000 steps were registered.

**Figure 2:**
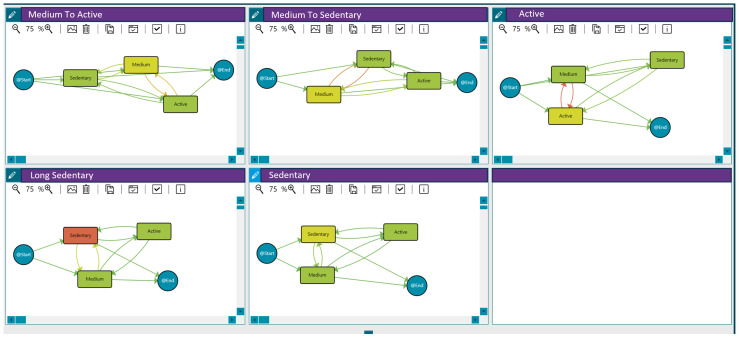
Activity behaviour groups for the 121 patients participating in the LifeChamps study with breast, prostate, or melanoma cancer diagnosis. Compared to green, an increase in an orange colour within the nodes indicates a longer median time spent in that stage. Similarly, a greater intensity of orange in the transitions signifies a higher number of cases associated with that transition, as detailed in the heat map legend.

Applying clustering techniques (see Section 3), we discovered groups of patients with similar activity behaviour, maximising differences with the rest of the groups to determine patterns. In this case, we used the threshold distance that maximises the differences between the traces reporting vulnerability and those that do not. Based on the activity level per day (sedentary, medium or active) and the transitions among days, five groups with different behaviours were discovered (see Figure 2):

- Medium to active: Patients with medium activity but a few active days.
- Medium to sedentary: Patients with medium activity but with some sedentary days.
- Active: Patients with an active pattern.
- Long sedentary: Patients with a long sedentary pattern.
- Sedentary: Patients with sedentary activity but with a few days in the medium or active categories.

These behaviour groups are represented in Figure 2. The different workflows summarise the sequence of activity levels, the duration spent in each state, and the frequency of transitions between them for each behaviour (with the time-varying risk variable being the number of steps per day). Moreover, Table 5 includes the distribution of patients in each group.

As outlined, the model represents patients’ activity based on their daily step count across three states: sedentary, medium, and active. Each state is depicted as a node, with arrows indicating transitions between states. Additionally, nodes labelled *@Start* and *@End* are included to signify the beginning and end points of the process, respectively. The model was enhanced with a heat map following the criteria and colour scheme collaboratively decided upon with the HCPs, ensuring the selected options effectively met their needs and preferences. These options are explained in each figure caption.

Then, we computed the RR, measuring the probability of reporting or not reporting vulnerability in the exposed groups. The computation results are included in Table 6. In each case, the exposed group corresponds with a concrete behaviour (non-exposed the rest), and the event would be reporting or not reporting vulnerability. We computed the RR for both the vulnerable and non-vulnerable groups, as we wanted to investigate the relative danger of sleep and activity patterns in reporting vulnerability and the relative protection of other sleep and activity patterns. For that reason, we computed RR for both groups. Groups with an RR exceeding 1 with a 95% confidence interval are highlighted in bold.

The results in Table 6 indicate that patients with a prolonged sedentary activity pattern have a 2.5 times higher risk of reporting vulnerability than those with other activity patterns, with a 95% confidence interval. This suggests a positive association between sedentary behaviour and the reported vulnerability outcome.

Continuing the analysis, we examined the potential association between cancer type (exposure) and vulnerability as the health outcome. We calculated the RR for the five cancer types included in the LifeChamps intervention, namely melanoma, prostate-curative, prostate-metastatic, breast-curative, and breast-metastatic, focusing on their relationship with reported vulnerability (see Table 7).

The Dynamic Risk Model for Vulnerability revealed that prostate-metastatic patients had more than three times the risk of reporting vulnerability compared to patients with other cancer types. Conversely, prostate-curative patients had 1.22 times the probability of not reporting vulnerability compared to patients with other cancer types. The reported vulnerability has a positive association with the prostate-metastatic cancer type. Conversely, the prostate-curative cancer type appears to be a protective factor against reported vulnerability in the study.

We also worked with health experts to discover and analyse other behaviours and affectations thanks to the data collected, such as body composition and heart rate variability. However, no other interesting and positive affectations were discovered for the Vulnerability model.

### 4.2. Anxiety/Depression Dynamic Risk Model

The Anxiety/Depression Dynamic Risk Model combined self-reported anxiety data from the PHQ-4 questionnaire with objective measures from the activity tracker. We considered that there was “No anxiety and depression risk” when none of the PHQ-4 questionnaires had a positive score (scores < 3). Conversely, we classified participants as having “Anxiety or depression risk (1)” when at least one PHQ-4 questionnaire had a positive score (score ≥ 3).

By incorporating self-reported and sensor-based data, this model aimed to provide a more comprehensive assessment of the participant’s mental health status and associated risk factors. The objective activity data from the tracker complemented the subjective questionnaire responses, enabling a deeper understanding of the relationship between physical activity and emotional well-being.

As explained in Section 3, we considered sleep to be inadequate or insufficient when the sleep efficiency was below 0.8, sleep to be normal or sufficient when the efficiency was between 0.8 and 0.89, and sleep to be beneficial or more than enough when it was 0.9 or over. Applying clustering techniques, we discovered three distinct groups of patients with similar sleep behaviour and some outliers. This analysis was based on the sleep efficiency per day and the transitions between sleep patterns across different days, considering the duration of the pilot study. In this case, we used the threshold distance that maximised the differences between the traces reporting anxiety/depression and those that did not. The three identified sleep pattern clusters can be characterised as follows:

- Insufficient Sleep: Patients in this group exhibited a consistently insufficient sleep duration throughout the pilot.
- High Sleep Dynamism/Insufficient with Dynamism: Patients in this group demonstrated a highly dynamic sleep pattern, with substantial variations in sleep efficiency across different days. Moreover, patients presented insufficient sleep on many days.
- Balanced Sleep: Patients in this group maintained a relatively balanced sleep efficiency pattern across the different days of the pilot.

**Figure 3:**
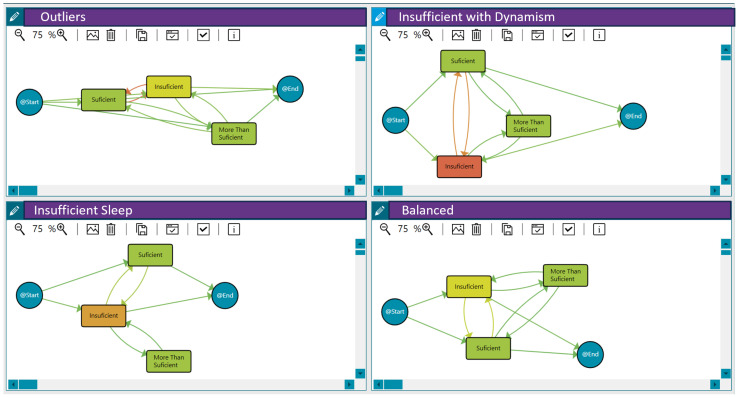
Sleep efficiency behaviour among the 121 patients in the LifeChamps study diagnosed with breast, prostate, or melanoma cancer. A shift from green to orange within the nodes indicates a longer median time spent in that sleep efficiency state. Similarly, a greater intensity of orange in the transitions signifies more patients moving between sleep efficiency states, while green indicates fewer patients making those transitions.

Figure 3 shows these behaviour groups for sleep efficiency. The different workflows represent the analysis of daily sleep efficiency using process mining techniques. The nodes and transitions provide insights into the patterns and transitions between sleep efficiency categories over time. Therefore, they summarise the sleep quality levels, the duration spent in each state/level, and the frequency of transition between them for each behaviour (the time-varying risk variable as the daily sleep efficiency). For example, the workflow for the insufficient with dynamism group highlights frequent fluctuations between insufficient and sufficient sleep efficiency. It reveals that individuals tend to spend more time in an insufficient state, suggesting a potential area for intervention to improve sleep quality. Furthermore, Table 8 includes the distribution of patients in each group. In addition to these three primary sleep behaviour clusters, the analysis also identified some outliers (48 patients) that did not fit neatly into the defined patterns.

This model illustrates patients’ sleep behaviour based on their daily sleep efficiency, categorised into three states: insufficient, regular or normal, and beneficial (more than sufficient). Each state is represented as a node, with arrows indicating transitions between them. Additionally, nodes labelled *@Start* and *@End* are included to denote the beginning and end points of the process.

The analysis revealed that certain factors, such as anxiety disorder, could be more likely to impact sleep efficiency. To further explore these relationships, we computed the RR of the identified sleep patterns concerning the reported anxiety and depression. The RR measure allowed us to quantify the probability of reporting anxiety and depression among the different sleep behaviour groups. The results of this computation are presented in Table 9.

The key finding of our analysis revealed that patients with highly dynamic sleep patterns—characterised by significant fluctuations in sleep efficiency across days—had a twofold increased risk of reporting symptoms of anxiety and depression compared to those in other sleep pattern groups. This finding indicates a strong association between unstable sleep behaviour and a heightened likelihood of experiencing anxiety and depression. By identifying these risk factors, we can develop targeted interventions to improve sleep quality and mental health outcomes for LifeChamps participants.

As detailed in Section 3, there is also a notable connection between anxiety levels and physical activity. To further explore this relationship, we calculated the relative risks (RR) associated with different activity patterns (see Figure 2) and their correlation with reported anxiety and depression. The results of this analysis are presented in Table 10.

In the context of the Anxiety/Depression Dynamic Risk Model, our analysis uncovered a significant relationship between activity patterns and reported anxiety levels. Consistent with the findings from the Vulnerability Dynamic Risk Model (see Section 4.1), patients who demonstrated prolonged sedentary behaviour were more than twice as likely to report anxiety symptoms compared to those in other activity groups. Notably, the analysis did not identify any specific activity patterns that served as protective factors against anxiety. This indicates that sedentary behaviour may be a considerable risk factor for elevated anxiety levels among LifeChamps participants.

Furthermore, no association was found between cancer type (exposure) and anxiety/depression outcomes when calculating relative risks. Based on the data collected, we collaborated with health experts to explore additional behaviours and factors, such as body composition and heart rate variability. However, our analysis did not reveal any significant positive influences related to the Anxiety/Depression model.

## 5. Discussion

Wearable devices have emerged as powerful tools in healthcare, enabling individuals to monitor their health and allowing providers to track this information proactively. The Fitbit Charge 4 and Withings Smart Scale Body+ exemplify how wearables enhance patient monitoring in clinical settings. Beyond data collection, the integration of AI significantly enhances the potential of these devices, facilitating in-depth analysis and supporting healthcare experts in making informed decisions.

Our approach does not aim to simply forecast disease occurrence in a strictly predictive sense but rather to model and analyse dynamic risk behaviours over time. This result is achieved by integrating data from wearable devices and self-reported questionnaires, providing objective and subjective insights into patient behaviour. We employed process mining techniques explicitly focusing on the interactive process mining (IPM) paradigm to achieve this.

Interactive process mining is a data-driven methodology that combines elements of machine learning like grammar inference techniques [42] or hidden Markov models and statistical techniques such as multistate models [43] with domain expertise to generate explainable models [14,44]. These models are co-created with HCPs, allowing them to interact with and refine the results based on their clinical knowledge. IPM emphasises transparency and interpretability, enabling clinicians to better understand the factors influencing patient behaviour. It is particularly valuable for mental health conditions, allowing for actionable insights to inform personalised treatment plans.

The innovation of this approach lies in its ability to provide a dynamic and interactive framework for analysing patient behaviours over time. By leveraging wearable data and self-reported measures, IPM models enable healthcare providers to uncover patterns in patient activity, sleep, and emotional well-being, which are critical for identifying and managing risks. Additionally, the explainable nature of these models ensures that HCPs can trust and effectively use the insights generated to improve patient outcomes.

This methodology significantly differs from traditional forecasting models, focusing on understanding and explaining patient behaviour in real time rather than solely predicting future states. By integrating clinical expertise into the modelling process, IPM fosters a collaborative and iterative approach to risk assessment, enhancing its applicability in real-world healthcare settings.

While some studies have utilised real-world data from wearable sensors, research on time-varying perspectives for determining risk factors in health outcomes remains limited. For example, ref. [18] applied machine learning algorithms for cardiovascular disease risk classification, showcasing AI’s potential in predictive modelling. Similarly, ref. [45] explores AI technologies in cancer prediction, noting that most research has prioritised improving diagnosis and prognosis through traditional medical histories. Numerous studies also examine AI’s role in cancer diagnosis and therapy [46,47].

Our work explores behavioural patterns derived from objective data collected via wearable sensors, examining their relationship with self-reported health outcomes to identify risky behaviours. By integrating real-world wearable data with subjective patient reports, we utilise process mining techniques to develop dynamic risk models for vulnerability and anxiety/depression in older cancer survivors. These models account for evolving, time-varying risk factors, offering a nuanced understanding of mental health over time.

Conducted within the LifeChamps study, our research involved data from 121 patients diagnosed with breast, prostate, or melanoma cancer across Greece, Sweden, Spain, and the UK. We engaged 36 HCPs in a co-creation framework, ensuring our outcomes directly address the real-world needs of patients and clinicians. This approach enhances the relevance of our findings and sets a precedent for future research in integrating wearable technology, AI, and patient-reported outcomes. Our findings include two dynamic risk models—one for vulnerability and another for anxiety/depression—analysed by computing relevant variables’ RR. This analysis investigates the impact of time-varying behaviours on these outcomes, providing actionable insights to enhance patient care and inform future interventions.

We developed two dynamic risk models that integrate self-reported data on vulnerability and anxiety/depression, measured through the PHQ-4 and VES-13 questionnaires via a smartphone app and objective data from wearable sensors. Specifically, we analysed daily activity levels (steps) and sleep efficiency using a Fitbit Charge 4, categorising activity as sedentary, moderate, or active and sleep efficiency as insufficient, regular, or beneficial. Using process mining techniques and clustering algorithms, we identified distinct patient groups based on their activity and sleep patterns, incorporating a time-varying risk perspective. For activity, we observed four behavioural patterns: active, sedentary, primarily sedentary with occasional moderate activity, and medium activity with some sedentary days. Regarding sleep efficiency, three patterns emerged: insufficient sleep, balanced sleep across categories, and a highly dynamic sleep pattern with frequent changes. The analysis revealed that prolonged sedentary behaviour significantly increases the risk of reported vulnerability, while dynamic sleep patterns are strongly associated with higher risks of reported anxiety among older adult cancer survivors. The study also found that older adult cancer survivors with metastatic prostate cancer are at a higher risk of reporting vulnerability compared to those with other cancer types, highlighting the need for targeted interventions for this group.

Identifying these time-varying risk factors can inform targeted interventions to improve vulnerability and anxiety outcomes for LifeChamps participants. Notably, no specific activity patterns emerged as being protective against anxiety, indicating that sedentary behaviour may significantly elevate anxiety levels among participants. Furthermore, no correlation was found between cancer type and anxiety/depression outcomes. Although other measures, such as body composition, did not yield significant associations, the findings suggest promising avenues for further research, especially with more extended studies and patients.

A key aspect of our study was the interactive and iterative approach employed in developing the dynamic risk models. Using co-design and co-creation methods, we aligned the models with clinical experts’ analytical needs, enhancing their understanding of the data and fostering meaningful dialogue between AI and clinicians. This collaboration transforms raw data into actionable insights that directly inform patient care. The iterative process further familiarises clinical experts with the analysis techniques, enhancing their confidence in utilising AI methodologies. This empowerment improves their ability to interpret results and nurtures a culture of innovation in clinical settings. Continuous engagement with data allows clinicians to refine their inquiries, leading to more targeted investigations and improved patient outcomes.

Integrating real-world and clinical data in oncology is essential for capturing patient perspectives and daily activities, allowing healthcare providers to tailor interventions more effectively. AI plays a critical role in this context by analysing large datasets from various sources, including wearables, to generate insights that guide clinical decision-making. Utilising process mining techniques enables a thorough examination of patient outcomes, helping to identify risk factors based on individual profiles. Incorporating wearables, such as the Fitbit Charge 4 and Withings Smart Scale Body+, into clinical monitoring significantly enhances patient engagement and equips HCPs with valuable tools for personalised care. As technology advances, the potential for wearables to transform clinical practice will likely increase, underscoring the need for ongoing research in this area. However, using wearable devices for clinical purposes also raises several important considerations that researchers and clinicians must address. These include ensuring data accuracy and reliability, fostering user compliance and engagement, addressing population bias, providing adequate contextual information, navigating data privacy and security concerns, and addressing regulatory challenges. While the wearable devices implemented in the LifeChamps study can enhance data collection in clinical trials, it is crucial for researchers to be aware of their inherent limitations and to use them appropriately based on specific clinical objectives. To maximise the value of wearable technology in clinical research, it is essential to tackle these challenges through thoughtful study design, comprehensive participant education, and robust data management practices. By addressing these factors, the integration of wearable devices can significantly contribute to improved patient outcomes and enrich the overall quality of care.

The data collected for this study are intended to demonstrate a relationship between the physical behaviour of the patient with data coming from multiple complementary sources: wearable devices (Fitbit Charge 4), in-home sensors, and validated questionnaires (PHQ-4 and VES-13). The wearables provide objective data on physical activity, sleep efficiency, and physiological variables. At the same time, the questionnaires capture subjective dimensions such as anxiety and vulnerability, offering a comprehensive view of dynamic risks. Additionally, the longitudinal approach (three months) allowed us to analyse temporal changes in behaviours and risks, and the diversity of the sample (121 patients from four countries with different cancer types and treatment stages) ensures the heterogeneity needed to validate the models. One limitation of this study is the exclusive focus on physical activity level and sleep quality as risk factors, while other potential predictors, such as medical history, psychological characteristics, or biomarkers, were not included. This choice was driven by considerations of future scalability and sustainability, as these indicators can be measured affordably and reliably using widely available technologies. Additionally, legal and ethical constraints across multiple clinical sites made the real-time collection of more sensitive data, such as medical histories or biomarkers, logistically unfeasible. Importantly, this study aimed to explore whether meaningful insights could be derived using only patient-reported data in a sustainable manner, aligning with the goals of the LifeChamps project.

This study is exploratory and aims to provide a proof of concept for the proposed methodology of modelling and analysing dynamic risk behaviours in older cancer survivors. While our sample size of 121 participants is limited, it was carefully selected to ensure diversity in cancer types (breast, prostate, and melanoma), treatment stages, and representation across multiple countries. The primary goal of this study is not to make generalisable statistical claims but to demonstrate the feasibility and potential of integrating wearable data, self-reported measures, and process mining techniques to uncover clinically actionable insights. Thus, there was no need to perform a simple size calculation [48].

We acknowledge that the sample size restricts the generalisability of our findings to broader populations. However, this exploratory approach provides an important foundation for future research involving larger, more representative cohorts. By focusing on proof of concept, we aim to validate our methodology and highlight its potential for application in real-world healthcare settings, paving the way for more comprehensive studies in the future.

In reflecting on the ethical concerns associated with medical data processing, it is crucial to recognise the multifaceted nature of these issues. Our study’s adherence to stringent ethical guidelines, including those set by the European Union, the Declaration of Helsinki, and other international standards, underscores our commitment to protecting patient data. The ethical approval obtained from multiple review boards and the implementation of a Data Protection Impact Assessment (DPIA) highlight our proactive approach to identifying and mitigating data protection risks. Furthermore, the establishment of a Joint Controller/Data Sharing Agreement among all involved parties ensures a collaborative effort in maintaining data confidentiality and integrity. These measures not only address potential ethical concerns but also reinforce the importance of transparency, patient autonomy, and trust in medical research.

## 6. Conclusions

Our analysis yielded valuable insights into how specific activity and sleep behaviours affect self-reported vulnerability and anxiety and how wearable sensors can provide data on these behaviours, analysed through AI techniques.

Despite this study’s contributions, several limitations warrant consideration. First, while patient-reported outcome measures (PROMs) are typically viewed as quantitative, they rely on subjective patient ratings regarding emotional states. This subjectivity can introduce variability, influenced by factors such as individual emotional conditions and the perceived effectiveness of interventions.

Another potential limitation of this study is the possibility of measurement errors associated with the wearable sensors used, particularly in tracking physical activity levels in older adult individuals. This issue arises from this study’s sustainability-driven approach, which prioritised the use of affordable and widely accessible devices. However, as this study was a feasibility study rather than a clinical trial, the level of accuracy provided by these devices was considered sufficient for this study’s objectives. Additionally, the interactive methodology (IPM) employed opens the possibility of applying advanced process mining data cleaning techniques, such as those described in [49], to collaboratively detect and address data inaccuracies in partnership with HCPs, thus enhancing the reliability of future applications.

Another limitation is that the RR values presented in Table 6, Table 7, Table 9 and Table 10 are based on small subsamples, sometimes comprising only 20–30 participants. This may result in wide confidence intervals and restrict the robustness of the conclusions. However, as this study is based on feasibility data, the objective was not to derive definitive clinical evidence but to explore the statistical behaviour of the population. The RR calculations provide preliminary insights that, when combined with the visual graphical models, allow HCPs to investigate patterns and generate ideas for personalised treatments. This exploratory approach highlights the potential for future research while acknowledging the limitations of the current findings.

This study involved 121 unique patients, allowing for diverse representation across demographics and cancer types and limiting generalisability. While the sample size meets current recommendations for feasibility studies [48,50], a larger cohort could strengthen the findings. Additionally, the LifeChamps study included four pilot sites, each with distinct healthcare contexts. Although all adhered to the same protocol, these differences may have affected participant perceptions and responses, introducing potential bias while enriching the insights from a transnational perspective.

While sufficient for initial observations, the three-month duration of the study may not capture the full range of fluctuating behaviours like activity and sleep, suggesting that more extended observation periods are needed for comprehensive analysis.

In summary, this study provides important insights into the dynamics of patient-reported outcomes and the role of wearable devices in monitoring variables affecting vulnerability and anxiety. However, the limitations related to subjectivity, sample size, and duration necessitate careful interpretation within their specific context. The preliminary results align with the existing literature, paving the way for future research.

Future studies should address these limitations by extending the observation period to capture fluctuations in activity and sleep behaviours throughout the cancer treatment journey. Incorporating additional data sources, such as electronic health records and physiological metrics or environmental factors, could provide a more holistic understanding of the factors influencing health outcomes, especially in older adult cancer survivors. Data collected during this study by the activity tracker included physiological data such as the heart rate variability or the breath rate. We worked with health experts to analyse these data, and although no interesting affectations were discovered in our case, more research is needed in this field.

This research underscores the potential for future studies to deepen our understanding of how activity and sleep behaviours impact self-reported vulnerability and anxiety, mainly through wearables and AI techniques. Longitudinal studies across various chronic conditions, including diabetes and heart disease, could reveal commonalities and differences in behavioural impacts on mental health. By building on this foundational work, future research can enhance the findings’ generalisability and practical relevance. It will facilitate targeted intervention design and foster integrated care approaches, ultimately improving treatments and outcomes for diverse populations managing chronic diseases. The methodology tested in this study was applied to a specific dataset, focusing on analysing concrete processes within the given population. As such, the findings are not intended to be generalisable at this stage. However, as more data become available and become statistically representative, the same methodology can be used to create models that represent broader populations. Additionally, the methodology is adaptable and reproducible across different contexts. By applying the study design to other populations, differences in behaviour and outcomes can be analysed, and deviations in processes can be identified using advanced process mining techniques. This adaptability, enabled by the visual behaviour analysis of the interactive process mining approach, is an advantage that supports future research in diverse settings.

## Figures and Tables

**Table 1:** Data sources of the LifeChamps intervention.

Device	Measures	Other Information
Activity tracker	Heart rate, heart rate variability, activity tracking, sleep monitoring, breath rate, skin temperature and SpO_2_	Patients were always asked to wear the wristband.
Smart scale	Weight and body composition	Patients were asked to use the smart scale once per week.
LOCS	Home sensors tracking ambulation and functioning: time and room	Four sensors were located in patients’ homes in the living room, kitchen, bathroom, and bedroom, plus one door sensor. Patients were asked to wear a tag at all waking times to differentiate from others living in the same house.
App	Patient profile information, Anxiety and Depression: Patient Health Questionnaire-4 (PHQ-4) [40], Frailty: The Vulnerable Elders Survey (VES-13) [41]	Patients were asked to fill out questionnaires once per month. The daily UV index was collected daily.

**Table 2:** Description of participants across pilot groups.

Country	Total	Age, X (SD)	Women (%)	Men (%)	Cancer Type
Greece	30	68.37 (7.85)	20 (66.7%)	10 (33.3%)	Breast, Prostate
Spain	50	70.02 (4.02)	27 (54%)	23 (46%)	Breast, Prostate
Sweden	21	73.57 (5.12)	5 (23.8%)	16 (76.2%)	Melanoma
Scotland	20	73.15 (5.44)	6 (30%)	14 (70%)	Breast, Prostate
Total	121	71.63 (5.93)	58 (47.9%)	63 (52.1%)	All

**Table 3:** Description of participants cont. Mode values are shown for each value.

Country	Background Education	Employment Status	Relationship Status	Living Arrangement	Support
Greece	University undergraduate	Retired	Married/ Partner	With spouse	Regularly
Spain	Primary school	Retired	Married/ Partner	With spouse	Regularly
Sweden	University undergraduate	Retired	Married/ Partner	With spouse	When needed
Scotland	University undergraduate	Retired	Married/ Partner	With spouse	Regularly/ When needed

**Table 4:** Description of HCPs participating in the co-creation process.

Variable	Responses	Total n = 36, %
Country (n,%)	Greece	10, 27.8%
	Spain	10, 27.8%
	Sweden	12, 33.3%
	Scotland	4, 11.1%
Gender (n, %)	Female	19, 52.8%
	Male	16, 44.4%
	Prefer not to say	1, 2.8%
Profession (n, %)	Medical Oncologist	11, 30.5%
	Geriatrician	2, 5.5%
	Clinical Nurse Specialist	1, 2.8%
	General Practitioner	5, 13.9%
	Acute Care Nurse	2, 5.5%
	Nurse	3, 8.3%
	Psychologist	1, 2.8%
	Urologist	3, 8.3%
	Radiation Oncologist	2, 5.5%
	Breast Surgeon	1, 2.8%
	Medical documentation	1, 2.8%
	Internal Medicine	1, 2.85%
	Other	3, 8.3%

**Table 5:** Description of participants across activity behaviour groups.

Activity Group	N. of Patients	% Total
Medium to active	42	34.7%
Medium to sedentary	25	20.7%
Active	24	19.5%
Long sedentary	19	15.7%
Sedentary	11	9.1%

**Table 6:** RR computation for vulnerability and activity behaviour groups. X (Y; Z), where X is the RR and (Y; Z) is the confidence interval. Groups with an RR exceeding 1 with a 95% confidence interval are highlighted in bold.

Activity Behaviour	No Vulnerable	Vulnerable
Medium to active	1.36 (0.70; 2.65)	0.74 (0.38; 1.43)
Active	1.58 (0.51; 4.89)	0.63 (0.20; 1.94)
Medium to sedentary	1.19 (0.45; 3.15)	0.84 (0.32; 2.23)
**Long sedentary**	0.39 (0.17; 0.88)	**2.58 (1.13; 5.85)**
Sedentary	0.60 (0.17; 2.11)	1.66 (0.47; 5.79)

**Table 7:** RR computation for vulnerability and cancer type. X (Y; Z), where X is the RR and (Y; Z) is the confidence interval. Groups with an RR exceeding 1 with a 95% confidence interval are highlighted in bold.

Cancer Type	No Vulnerable	Vulnerable
Melanoma	1.14 (0.94; 1.38)	0.43 (0.06; 2.89)
**Prostate-Curative**	**1.22 (1.07; 1.39)**	0.20 (0.03; 1.42)
**Prostate-Metastatic**	0.59 (0.33; 1.04)	**3.28 (1.62; 6.65)**
Breast-Curative	0.93 (0.74; 1.17)	1.33 (0.59; 3.02)
Breast-Metastatic	1.02 (0.71; 1.48)	0.90 (0.14; 5.58)

**Table 8:** Description of participants across sleep behaviour groups.

Sleep Group	N. of Patients	% Total
Insufficient with Dynamism	35	28.9%
Insufficient Sleep	22	18.2%
Balanced	16	13.2%

**Table 9:** RR computation for anxiety and sleep behaviour groups. X (Y; Z), where X is the RR and (Y; Z) is the confidence interval. Groups with an RR exceeding 1 with a 95% confidence interval are highlighted in bold.

Sleep Behaviour	No Anxiety	Anxiety
Insufficient sleep	1.46 (0.45; 4.76)	0.68 (0.21; 2.23)
**Insufficient sleep with dynamism**	0.49 (0.30; 0.83)	**2.02 (1.21; 3.09)**
Balanced sleep	1.41 (0.32; 6.18)	0.71 (0.16; 3.11)
Outliers	1.34 (0.80; 2.25)	0.75 (0.45; 1.25)

**Table 10:** RR computation for anxiety and activity behaviour groups. X (Y; Z), where X is the RR and (Y; Z) is the confidence interval. Groups with an RR exceeding 1 with a 95% confidence interval are highlighted in bold.

Activity Behaviour	No Anxiety	Anxiety
Medium to active	1.88 (0.94; 3.75)	0.53 (0.27; 1.06)
Active	0.63 (0.30; 1.32)	1.60 (0.76; 3.37)
Medium to sedentary	1.25 (0.51; 3.06)	0.80 (0.33; 1.95)
**Long sedentary**	0.43 (0.19; 0.97)	**2.32 (1.03; 5.26)**
Sedentary	0.84 (0.24; 2.96)	1.20 (0.4; 4.24)

## Data Availability

The data presented in this study are available on request from the corresponding author due to ethical restrictions.

## Abbreviations

The following abbreviations are used in this manuscript:

AI	Artificial intelligence
BMI	Body Mass Index
ECG	Electrocardiogram
EHRs	Electronic health records
GAD-2	Generalized Anxiety Disorder 2-item
HCPs	Healthcare professionals
IPIs	Interactive process indicators
IPR	Interactive pattern recognition
IPM	Interactive process mining
IQR	Interquartile range
LC	LifeChamps
Spo2	Oxygen saturation
PHQ-4	Patient Health Questionnaire-4
PI	Process Indicator
PROMs	Patient-reported outcome measures
PM	Process mining
QoL	Quality of life
QTC	Quality Threshold Clustering
REM	Rapid eye movement
SIOG	International Society of Geriatric Oncology
PSA	Prostate-specific antigen
SpO2	Oxygen saturation
UV	Ultraviolet
VES-13	Vulnerable Elders Survey-13
